# Comparative analysis of muscle synergies in gait of stroke patients and healthy controls

**DOI:** 10.3389/fnhum.2025.1601147

**Published:** 2025-07-16

**Authors:** Dimitrios-Sokratis Komaris, Dimitrios Tsaopoulos, Konstantinos Risvas, Spiros Nikolopoulos, Vasilios Baltzopoulos, Constantinos Maganaris, Ioannis Kompatsiaris

**Affiliations:** ^1^Centre for Research and Technology Hellas, Information Technologies Institute, Thessaloniki, Greece; ^2^School of Sport and Exercise Sciences, Faculty of Science, Liverpool John Moores University, Liverpool, United Kingdom

**Keywords:** stroke, gait, electromyography, EMG, locomotion, rehabilitation, muscle synergy, NNMF

## Abstract

**Introduction:**

This study analyzed muscle synergies in subacute stroke patients’ gait, comparing paretic and non-paretic limbs with healthy individuals, and explored the structure and temporal activation of synergistic muscle activation patterns.

**Methods:**

Muscle synergies were computed from EMG data of 12 muscles in 138 able-bodied adults and 50 stroke survivors using non-negative matrix factorization, analyzing 350 control strides, 319 paretic strides, and 337 non-paretic strides. Temporal activation coefficients of the muscle synergies between groups of strides were also compared using cross-correlation analysis.

**Results:**

The extracted muscle synergies were consistent in composition across all groups of control and stroke subjects, with four synergies identified in gait cycles on average. The comparison of the synergies’ temporal activation returned indexes (r) ranging from 0.60 to 0.74, with differences existing in the duration and timing of the activations of the hip flexors and knee extensors, dorsal flexors, and plantar flexors.

**Discussion:**

Our findings suggest a certain degree of preserved motor function in stroke patients’ gait, even in the presence of recent hemiparesis, but with evident alterations in the synergies’ temporal activation. Stroke rehabilitation by targeting abnormal muscle synergy activations may help shape personalized treatment plans.

## 1 Introduction

In Europe, stroke is the second leading cause of death and the primary cause of disability, with associated costs reaching approximately €45 billion (Timmis et al., 2017). In 2019, there were over 13.7 million new reported strokes globally, and one in four people over the age of 25 will have a stroke in their lifetime (Kim et al., 2020). Following discharge from the stroke unit, post-acute patients treated early in intensive rehabilitation facilities show better functional outcomes and higher rates of returning to community living compared to those in general ward or long-term care hospitals (Hong et al., 2019; Wang et al., 2011). Moreover, patients with stroke-related hemiparesis demonstrate a prolonged sensitivity to treatment that extends well beyond the traditional 3–6-month “critical window” of heightened neuroplasticity following a stroke (Ballester et al., 2019), emphasizing the need of a continued and adaptable rehabilitation approach. A key challenge remains in determining how to tailor therapeutic interventions [e.g., functional electrical stimulation, or FES for short; also refer to Ferrante et al. (2016)] at an individual level by adjusting the intensity and type of activities to maximize functional recovery. Integrating such biomarkers and their dynamic changes into rehabilitation models could enhance the personalization of recovery plans, potentially reducing disability and improving quality of life. Muscle synergy analysis (d’Avella et al., 2003) may serve in this regard, by assessing the underlying coordination and gait deficits of stroke survivors during their rehabilitation, providing deeper pathophysiological insights and helping to optimize rehabilitation strategies that target individual changes in neuromuscular coordination.

Muscle synergy analysis has been used to inspect coordination in a plethora of complex motor tasks, and even though it remains unclear whether these muscle synergies in fact originate from the central nervous system, their potential clinical value in novel stroke rehabilitation approaches is well-supported (Hong et al., 2021). The method employs activation profiles from a large number of muscles measured by means of electromyography (EMG) during a motor task, and proceeds in a matrix factorization resulting into weighted groups of muscles, called muscle synergies (from this point forward denoted as W), each characterized by specific time-varying activation profile (denoted as C) (d’Avella et al., 2003). These muscle synergies (also occasionally referred to as modules) can then be interpreted as fixed motor programs that are dependent on the performed movement. For example, in healthy adult walking, four distinctive muscle synergies are often observed [i.e., W1: hip abductors and hip/knee extensors; W2: plantar flexors; W3: dorsal flexors; W4: Hamstrings (Van Criekinge et al., 2020)] each predominantly activated at different phases of the gait cycle (e.g., muscle synergy W3 has a typical activation profile, referred to as C3, that shows a peak during late stance). Combinations of different muscle synergies and their corresponding temporal activation profiles can be used as building blocks to reconstruct the muscle patterns involved in any motor task, effectively capturing the complexity of human motor behavior.

In human gait, specific muscle synergies have been linked to distinct phases of the gait cycle (Barroso et al., 2017). For instance, a synergy mainly composed of the ankle plantarflexors is typically present during the late stance in healthy individuals (Clark et al., 2010; Van Criekinge et al., 2020). Muscle synergy analysis has also demonstrated sufficient sensitivity to detect variations in the number and temporal activation of synergies across different locomotory tasks, such as perturbed walking (Chvatal and Ting, 2013), moving with varying foot strike patterns (Nishida et al., 2017) or different speeds (Kibushi et al., 2018). Moreover, the number of synergies appears altered in the gait of individuals with musculoskeletal-related diseases or disorders, such as cerebral palsy (Steele et al., 2015), Parkinson’s (Rodriguez et al., 2013) and multiple sclerosis (Janshen et al., 2020). Therapeutic interventions have also been found to influence muscle synergy structure or improve existing abnormal strategies, as demonstrated in hemiplegic patients undergoing physical therapy (Kogami et al., 2018) and acute post-stroke patients using robotic therapy devices (Dipietro et al., 2007; Tan et al., 2018). These findings indicate that muscle synergy analysis may help in targeting muscle groups related to specific conditions or phases of the gait cycle –aiming, for example, to improve gait stability or foot clearance.

Although several studies investigated the nature of muscle synergies in stroke patients, these efforts principally focused on the upper extremities [e.g., (Israely et al., 2018; Roh et al., 2013, 2015)] or, when considering the lower body, on the influence of therapeutic interventions post-stroke, while targeting the function of the paretic side only [e.g., (Ferrante et al., 2016; Tropea et al., 2013)]. The present work assessed the muscle synergy composition of stroke patients in overground waking with self-selected speed, examining separately the gait cycles of paretic and non-paretic limbs alike, and as compared to healthy age-matched individuals. Having an individualized look at the behavior of the paretic and not-paretic limbs may provide additional insight in the motor behavior of hemiplegia or the development of personalized rehabilitation strategies for stroke management. For example, post-stroke hemiparesis is often accompanied by a variety of compensatory mechanisms from the non-paretic limb to improve overall motor performance [e.g., to provide greater propulsion or support (Raja et al., 2012)] that may lead to different muscle synergy compositions. Additionally, muscle synergy analysis of the paretic side can also facilitate the future development of rehabilitation interventions that target each leg separately [e.g., the development of FES controllers for the paretic limb based on muscle synergy analysis; see (Ferrante et al., 2016) for more information] that may also have an indirect effect on the reduction of compensatory mechanisms on the contralateral limb.

In alignment with the above, we further investigated whether the temporal activation of the muscle synergies (for both the paretic and non-paretic sides) is invariant across the strides of our cohort of stroke patients and healthy subjects, by comparing the temporal activations of each stride individually in the studied dataset. Given that recent research on muscle synergies suggests targeting specific neuromuscular patterns could optimize personalized rehabilitation (Hong et al., 2021), a better understanding of the muscle coordination during walking may help in improving assessment and facilitate bespoke individualized treatments.

## 2 Materials and methods

### 2.1 Participants and data collection overview

We have utilized an open-access full-body motion capture gait dataset (Banks et al., 2017) consisting of 138 able-bodied adults (aged 21–86 years; 73 females; 74 ± 15 kg body mass; 1684 ± 103 mm height) and 50 age-matched stroke survivors (aged 19–85 years; 16 females; 72 ± 14 kg; 1705 ± 80 mm). The dataset was last accessed in September 2024. Patients had a confirmed ischemic (*n* = 39) or haemorrhagic (*n* = 11) stroke within 5 months prior to data collection, placing them, to a reasonable extent for the purposes of this analysis, to the same recovery stage. Stroke survivors had a mean time since stroke onset of 53 days (SD = 19), with 33 presenting with right-hemispheric lesions, and 17 with left-hemispheric lesions. Stroke subjects were also evaluated with a Functional Ambulation Category test (mean score = 3, SD = 1, min-max = 2–5), Trunk Impairment Scale (mean score = 14, SD = 3, min-max = 7–20), and a Tinetti POMA (mean score = 19, SD = 6, min-max = 6–28). Kinematic data were recorded at 100 Hz using the Plug-in Gait (PiG) full-body biomechanical model. Muscle activity from 13 muscles on the trunk and lower limbs was recorded using a 16-channel surface electromyographic (EMG) system recording at 1000 Hz as per the SENIAM recommendations (Gizzi et al., 2011) and confirmed by selective muscle contractions. Sensors were placed on the following muscles bilaterally (left and right for the control subjects, denoted as: L/R; paretic and non-paretic for stroke survivors, denoted as: P/N): rectus femoris (RF), vastus lateralis (VL), biceps femoris (BF), semitendinosus (ST), tibialis anterior (TA), and gastrocnemius (GAS). Overall, a minimum of six overground strides during steady-state walking, at self-selected cadence (1.21 ± 0.17 m/s on average for the controls; 0.49 ± 0.29 m/s for the stroke patients) were recorded per participant. Kinematic data were filtered using a 4th-order low-pass Butterworth filter with a cut-off frequency of 10 Hz. EMG signal normalization and other post-processing techniques will affect the quality of the signal for clinical purposes (Burden and Bartlett, 1999). In the employed dataset, all EMG data were normalized to the maximum found value over all the available strides of each subject per muscle (Banks et al., 2017; Safavynia et al., 2011). EMG signals were additionally band-pass filtered (at 10–300 Hz), rectified, and then smoothened with a low-pass finite-impulse filter with a 20 Hz cutoff frequency [similar to Barroso et al. (2017), Chvatal and Ting (2013), Kautz et al. (2011)]. Kinematic and EMG data were also time-normalized to 1000 points per stride.

### 2.2 Muscle synergy analysis: synergy composition (W) and temporal activation profiles (C)

Synergy analysis was performed on all the available strides of the pre-processed EMG data of the dataset, and the average number of muscle synergies per group (control, non-paretic, paretic) and their corresponding temporal coefficient was extracted similar to Russo et al. (2024). Paretic and non-paretic sides of the patient group were determined based on brain lesion location (left and right) primarily affecting the contralateral side of the body. In total, we have included in the analysis 350 strides from the control subjects (left and right limbs jointly), 319 strides from the stroke survivors’ paretic side, and 337 strides from the non-paretic side. Muscle synergies were extracted from EMG signals by means of the most frequently used evaluation algorithm (Boccia et al., 2018), called non-negative matrix factorization (NNMF), adopting the procedures outlined by Russo et al. (2024). The matrix input to the muscle synergy extraction was a *s* × (*m* × 1000) matrix, where *m* is the number of muscles (i.e., six muscles per limb in this analysis, the GAS_*L/R*_, RF_*L/R*_, VL_*L/R*_, BF_*L/R*_, ST_*L/R*_, TA_*L/R*_ for the control subjects), *s* is the number of strides per group, while all EMG data are normalized to 1000 points per stride. This method decomposes the EMG signals of muscles into a number of muscle synergies, each characterized by a spatial synergy matrix (W) and a set of temporal activation coefficients (C). Matrix W embodies muscle activation patterns, where each column represents a synergy, or simply, a group of muscles that co-activate in a coordinated manner along with the weighted contribution of each muscle to a given synergy (i.e., with each synergy typically dominated by the activity of a subgroup of the muscles considered). Conversely, each row of the C matrix contains a weight that represents the temporal activation of each synergy over time, or in this analysis, a gait cycle (i.e., in which portion of the gait cycle a synergy W is mostly active). Together, W and C matrices enable the reconstruction of the original EMG signals (D): D≈WC. The range of potential number of synergies to extract and the number of iterations of synergy extractions for each number of synergies were set to *N* = [1:6] and 10, respectively. In other words, the process searched for possible solutions for up to 6 muscle synergies per group, i.e., the maximum number of muscles considered. For each muscle synergy solution N, an optimization gradient descent algorithm assigned random values between (??) in the weights of the spatial synergy W and coefficient C matrices, and used an iterative process of 10 iterations to optimize these weights based on the reconstruction quality of each iteration. The optimum number N of synergies per group (i.e., control, paretic, and non-paretic) was selected based on the reconstruction quality (R^2^) curve for the different numbers of extracted synergies using a threshold of 0.8, as per the recommendations by Russo et al. (2024). Finally, the number and muscle weights of spatial synergies (W) of each group, and the average associated temporal activation coefficients (C) per gait cycle were extracted as bar-charts and time-series graphs for visualization. For more information on the details of the employed optimization NNMF algorithm and methodological aspects for the muscle synergy extraction, please refer to the associated publication (Russo et al., 2024).

### 2.3 Cross-correlation analysis

**FIGURE 1:**
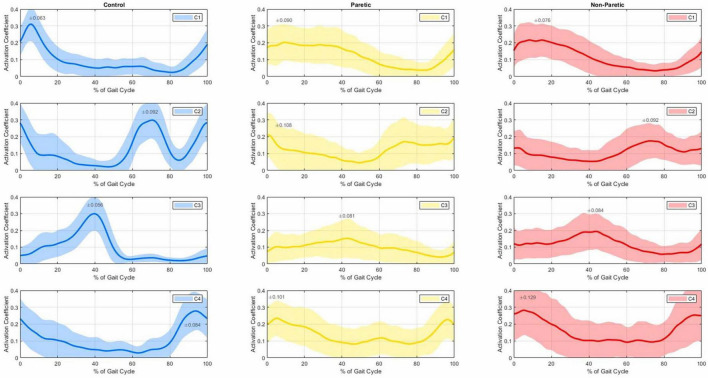
Average temporal activation coefficients (C1 top row to C4 bottom row) with 1 SD (shaded regions) of the control group (**left** 3 panels), paretic (**middle** 3 panels) and non-paretic groups (**right** 3 panels), as a% of the gait cycle.

On top of the averaged muscle synergy analysis per group, the activation coefficients C between each individual stride of the control, paretic, non-paretic sides (i.e., the individual coefficients C of each stride in this part of the analysis, and not to be confused with the averaged coefficients per group as later presented in Figure 1) were then compared using cross-correlation analysis [similar to Boccia et al. (2018), Chvatal and Ting (2013), Tropea et al. (2013)]; the vectors from the activation coefficients C matrices were evaluated using their scalar product normalized by the product of their norms (i.e., cosine similarity), emphasizing vector shape over amplitude. Mean correlation coefficients (*r*) were calculated across all possible pairs to summarize the overall similarity between the comparisons made (i.e., paretic vs non-paretic, control vs paretic, and control vs non-paretic strides), with higher values indicating greater similarity.

## 3 Results

### 3.1 Muscle synergy composition (W)

**FIGURE 2:**
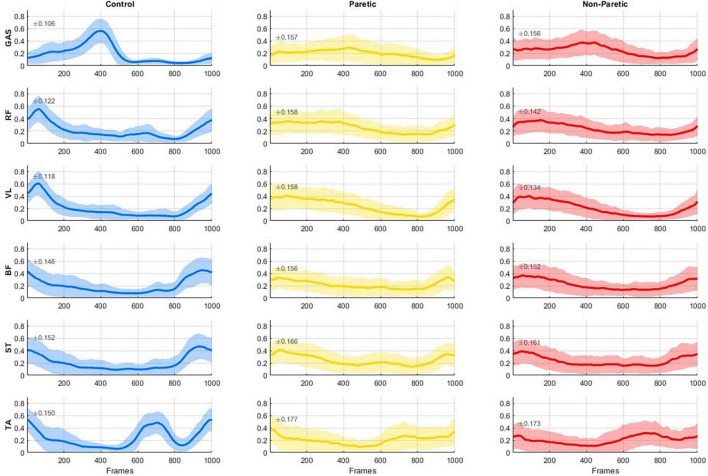
Group-averaged electromyography (EMG) activity (solid line) with 1 SD (shaded regions, SD magnitude is also noted on each panel) of six muscles (GAS, gastrocnemius, RF, rectus femoris; VL, vastus lateralis; BF, biceps femoris; ST, semitendinosus; and TA, tibialis anterior) reported for the healthy controls **(left)**, the paretic side of stroke patients **(middle)**, and the non-paretic side of stroke patients **(right)**. EMG activity is normalized to the maximum value across all strides of each subject for every muscle. All data were time-normalized to 1000 points per stride.

The averaged EMG activity of the considered six muscles for each group (control, paretic, non-paretic sides) are reported in Figure 2; in line with previous reported data, lower limb muscle activation showed typical involvement during conventional walking in the control subjects (Boccia et al., 2018), and large variability (shaded regions in the graphs) in the strikes of the stroke subjects (Banks et al., 2017; Mizuta et al., 2022).

**FIGURE 3:**
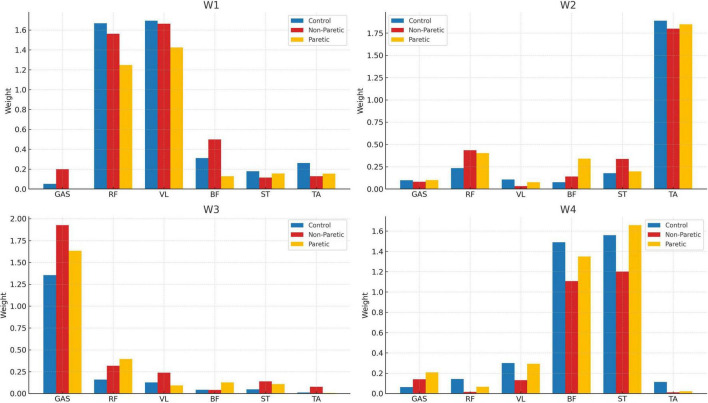
Muscle weightings per synergy (W1–W4) of the control (blue), non-paretic (red), and paretic (yellow) groups.

The number and structure of the extracted muscle synergies was consistent across all groups, with four muscle synergies (W1 to W4) found overall in the strides of the control subjects (in blue, Figure 3), and the non-paretic (in red) and paretic sides (in yellow) of the stroke patients. For reference, the R^2^ values for the optimum synergy solutions were close among all groups, with values ranging between 0.315–0.338, 0.531–0.565, and 0.705–0.751, for the 1-, 2-, and 3-cluster solutions, respectively. Here, the spatial synergy matrix (W) of the four-synergy solution (W1-W4) is given in bar charts (Figure 3), with the vertical axes corresponding to the muscles composing each synergy, and the horizontal axes corresponding to the weighting of each muscle that compose each synergy. In every group, muscle synergies were predominantly associated with the activation of the hip flexors (RF: rectus femoris) and knee extensors (VL: vastus lateralis) in the W1 synergy (see Figure 3, top three panels); the dorsal flexors (TA: tibialis anterior) in W2; the plantar flexors/knee extensors (GAS: gastrocnemius) in W3; and finally, the hamstrings (BF: biceps femoris and ST: semitendinosus) in W4.

### 3.2 Temporal activation profiles (C)

Figure 1 shows the average temporal activation coefficients of the control, paretic and non-paretic groups normalized to 100% of the gait cycle for each muscle synergy. Hip flexors and knee extensors of the first muscle synergy (W1) show a temporal activation (C1) seemingly occurring from terminal swing (approximately at 90% of the gait cycle; see C1 graphs, Figure 1) to loading response for the control group (with a peak at ≈10% of the gait cycle; left panel, top row), and up to terminal stance for the paretic and non-paretic sides (30%–40% of the gait cycle; middle and right panels, top row). The dorsal flexors of the W2 synergy appear to be primarily activated (C2) during heel strike (with a first peak appearing on the graph at the beginning of the cycle) and again at initial swing (with a second peak, roughly, at 70% of the cycle); the activity of the dorsal flexors of the controls appears to sharply drop at ≈90% of the gait cycle (C2; 2nd row, left panel), a pattern which is not evident to the same extent in the synergy’s temporal activation of the paretic and non-paretic groups (C2; 2nd row, middle and right panels). Synergy W3 of the plantar flexors’ muscles is predominantly activated at the 40% mark of the gait cycle (C3), which typically corresponds to the terminal stance of the gait. Even though plantar flexors do not show any evidence of activity during the swing phase in the controls (≈55%–100%; 3rd row, left panel), their activation pattern persist until mid-swing for the non-paretic group, and even longer for the paretic group (≈60%–90%; 3rd row, middle and right panels). Finally, the temporal activation of the hamstring muscles (C4) shows peaks of activation after the mid swing (at ≈90% of the cycle here) into the early stance of the next gait cycle (roughly at 10%, which generally corresponds to the heel strike), again, with a prolonged activation in the paretic and non-paretic limbs.

### 3.3 Cross-correlation analysis

To further investigate this degree of heterogeneity between the temporal activation coefficients of all groups (see Figure 1) we have additionally computed the cross-correlation coefficients between the signals of all the individuals in each group, revealing a fair degree of homogeneity in the shape of the time series between all comparisons (Table 1). The temporal activation of the four muscle synergies between paretic and non-paretic strides showed fair similarity with cross-correlation coefficients *r* ranging from 0.604 to 0.736; similarly, strides did not differ between control and paretic (*r* from 0.602 to 0.665), nor between control and non-paretic sides (0.645 and 0.722). The higher index of similarity (*r* = 0.736) was found in the activation of the muscles of the W1 strategy (i.e., C1 of the hip flexors and knee extensors) in the comparison between paretic and non-paretic limbs of the stroke patients, whereas the lowest index (*r* = 0.602) was observed in the comparison of the W2 synergy between the control and paretic side of the stroke patients. For reference, in studies analyzing muscle synergies, similarity indexes *r* between 0.6 and 0.8 often denote a comparable degree of homogeneity (Chvatal and Ting, 2013; Gizzi et al., 2011), while lower values are often interpreted as indexes of dissimilarity between synergies and their activation coefficients. In our analysis, similarity indexes were lower, in every comparison, between control and paretic limbs (Table 1).

**TABLE 1:** Cross-correlation coefficients (R) between the activation coefficients (C) of the control (*n* = 350 strides), paretic (*n* = 319 strides), non-paretic (*n* = 337 strides) groups.

Temporal activation coefficients	Paretic/non-paretic	Control/paretic	Control/non-paretic
C1	0.736	0.665	0.722
C2	0.604	0.602	0.645
C3	0.680	0.633	0.695
C4	0.687	0.648	0.651

## 4 Discussion

Research on muscle synergies while walking in stroke patients, whether overground or on a treadmill, remains limited. Few studies have simultaneously examined both healthy individuals and the paretic and non-paretic sides of stroke survivors alike. Notably, Gizzi et al. (2011) evaluated EMG signals of 10 stroke patients and 10 healthy controls, whereas Clark et al. (2010) performed a similar analysis with 55 adults with post-stroke hemiparesis and 20 healthy adults. Our study builds on and extends this body of work by evaluating gait patterns in a larger cohort of 50 stroke survivors and 138 able-bodied adults (Figure 3), while also examining the level of similarity between the temporal activation coefficients of the studied population using cross-correlation analysis.

### 4.1 Muscle synergy composition (W)

When analyzing the number of extracted muscle synergies, and while considering six muscles per limb, we consistently identified an average of four muscle synergies across all groups during overground walking with self-selected speed (Figure 3). It is generally accepted that a higher number of synergies correlates with more integrated motor function, while merged synergies are associated with compensatory mechanisms (Barroso et al., 2017; Safavynia et al., 2011). Existing literature also suggests that the number and composition of muscle synergies in subacute stroke patients varies depending on the specific muscles chosen for analysis and their total number. As a point of reference, a systematic review by Van Criekinge et al. (2020) found that the number of synergies in walking for the control and non-paretic sides is generally four, whereas for the paretic side, studies reported between two and five synergies with four synergies on average (Barroso et al., 2017; Safavynia et al., 2011). Thus, our findings of four synergies in all groups align well with previously reported results in overground walking [for example, refer to Coscia et al. (2015), Gizzi et al. (2011), Kautz et al. (2011)]. However, these findings extend only to subacute stroke patients, and they are subject to the time since stroke onset (TSSO). For example, it is well reported that the number and structure of muscle synergies in walking changes during the rehabilitation process and as patients exhibit recovery in terms of muscle strength and range of motion (Cheung et al., 2012; Hashiguchi et al., 2016). Longitudinal studies also reported that muscle synergies on the paretic side increased from two during admission to up to four after 2 months of rehabilitation training (Ebihara et al., 2023).

However, analyzing more muscles does not necessarily lead to the extraction of a greater number of synergies – that is, compared to the six that were used in the present work. For instance, Clark et al. (2010) measured the activity of eight muscles (i.e., tibialis anterior, medial gastrocnemius, rectus femoris, semitendinosus, biceps femoris, vastus medialis, soleus, and gluteus medius; bold denotes mutual muscles with the present study) and found four synergies for the control and non-paretic sides, while reporting two synergies in 45% of the paretic strides and three synergies in 36%. Similarly, Gizzi et al. (2011) studied a set of sixteen muscles and a subset of seven (i.e., tibialis anterior, gastrocnemius, rectus femoris, biceps femoris, vastus lateralis, soleus, and gluteus maximum) and consistently observed four synergies in both healthy controls and stroke survivors (paretic and non-paretic sides) regardless of the considered number of muscles. Finally, Routson et al. (2013) also employed eight muscles (tibialis anterior, medial gastrocnemius, rectus femoris, rectus femoris, semitendinosus, vastus medialis, soleus and gluteus medius) and reported three to four muscle synergies for the stroke group. In terms of methodological considerations for the better implementation of the method for stroke rehabilitation, our findings suggest that the variation in the number of muscle synergies is more likely attributed to the specific muscles selected (or omitted) rather than the overall number of muscles analyzed [a comparable conclusion was reached in Van Criekinge et al. (2020)]. For example, muscles such as the gluteus medius and soleus that were not included in the present study may explain variations in the number and composition of muscle synergies among studies, and provide a more comprehensive understanding of proximal joint control, particularly in stroke-specific gait impairment. It is also important to note here that ipsilateral gluteus medius is a muscle particularly hindered both in latency and activation in stroke patients (Kirker et al., 2000), and ipsilateral soleus is an important contributor to increasing walking speed and higher functional walking status in stroke subjects (Hall et al., 2011). Additionally, walking speed appears to be another parameter that directly affects the activity of such muscles in walking, and also the exhibited number of muscle synergies (Safavynia et al., 2011). Kibushi et al. (2018) further investigated the average number of synergies across different walking speeds, and reported four synergies for walking speeds of approximately 0.55 m/s, and five synergies for speeds higher than 1.25 m/s. As a reference, control subjects in this study walked with an average speed of 1.21 m/s, and stroke patients with a concise speed of 0.49 ± 0.29 m/s which is comparable to the walking speed reported in the stroke cohort of Gizzi et al. (2011) (0.53 ± 0.25 m/s) and Clark et al. (2010) (0.58 ± 0.26 m/s).

### 4.2 Cross-correlation analysis

Apart from the number and structure of muscle synergies, differences in synergy temporal activation will affect the timing and strength of motor tasks (Safavynia et al., 2011). To further explore this, we have additionally analyzed the degree of homogeneity among the temporal activation coefficients of each group (Table 1) and deducted relatively similar cross-correlation coefficients ranging between 0.60 and 0.74. Among all comparisons, the lowest coefficients were observed in the comparisons between control and paretic limbs. However, in stroke participants, motor impairment on the paretic side requires compensatory activation on the non-paretic side to maintain gait functionality (Raja et al., 2012). Therefore, the paretic and non-paretic sides of each participant are functionally dependent. As a result, and as a limitation of this analysis, part of the similarity observed in cross-correlation analyses between the paretic and non-paretic sides may be, to some extent, biased due to the within-subject similarity of the analyzed gait cycles.

### 4.3 Temporal activation profiles (C)

Evident differences and latencies exist in the morphology of the synergies’ temporal activations between the studied groups (Figure 1). Firstly, hip flexors and knee extensors (C1) appear to be activated for a prolonged period in the paretic and non-paretic limbs and for the entirety of the stance phase, and as compared to the control limbs that remain briefly active for the heel strike only (see C1, Figure 1). This prolonged activation of hip flexors and knee extensors during stance is commonly exhibited in stroke patients and is largely due to compensatory muscle synergies, spasticity in the gluteus maximum muscles and quadriceps, and altered neural control, which can impact efficient movement (Li et al., 2018). The second synergy (W2) includes the dorsal flexors, activated during heel strike and mid-swing (C2), showing a noticeable drop followed by a sharp rise at ≈90% of the gait cycle (i.e., prior to heel strike) in the control subjects which are not present in the gait of the stroke patients. As a reference, dorsiflexors are the muscles that raise the ankle and foot, and dorsiflexors’ activity is often reduced or delayed during the terminal swing phase of walking in stroke patients, leading to many stroke survivors experiencing “foot drop” and inappropriate foot clearing (Ng and Hui-Chan, 2012). The third synergy (W3) is mainly composed of the gastrocnemius (a plantar flexor and knee flexor muscle), which is activated only during stance for the controls, but similarly remains (partially) active even up to the initial swing for the stroke patients (C3). Finally, the fourth synergy (W4) involves the hamstrings (knee flexors), with activation from mid-swing into early stance for all groups (C4), with a laten activation in the gait of the paretic and non-paretic limbs (Figure 1) during the early stance phase compared to healthy individuals. This compensatory mechanism could be linked to an abnormally heavy reliance on hamstrings to flex the knee during the early stance phase in stroke patients. Our findings are also in agreement with the four-muscle-synergy composition reported by Van Criekinge et al. (2020) and Clark et al. (2010) which describe similar muscle groups and synergy activation: (i) hip abductors and hip/knee extensors activated at early stance, (ii) dorsal flexors during early swing, (iii) plantar flexors in late stance, and (iv) hamstrings in late swing and early stance. In this composition, the Glutei Medius and Maximus are incorporated into the first synergy of the hip abductors and hip/knee extensors.

### 4.4 Overview, limitations, and perspectives for future work

Our findings indicate that despite recent hemiparesis, stroke survivors retain a degree of preserved motor function, as evidenced by the consistent synergy composition across both paretic and non-paretic limbs. Rehabilitation efforts and bespoke treatments could focus on improving the prolonged or delayed temporal activation of these preserved synergies, particularly in the dorsal flexors (see C2 in Figure 1 with an *r* of 0.602 in Table 1) and plantar flexors (C3, *r* = 0.633) to enhance gait stability and reduce such compensatory mechanisms. A key limitation of our analysis is the relatively small set of six muscles used to assess self-selected low-speed walking. However, as long as the dataset includes core muscles associated with the building synergies of the gait cycle–such as hip abductors/extensors, knee extensors, plantar flexors, dorsiflexors, and hamstrings– low-speed walking tends to cluster into four muscle blocks. Expanding the analysis to include additional muscles, such as the gluteus medius and soleus, may still yield the same number of synergies but it can reveal important variations in their structure, timing, and clinical interpretation. These differences are crucial for enhancing the clinical relevance of the method. Identifying such variations is key to detecting pathological delays or activation patterns, which can then be addressed through targeted physiotherapy (Hong et al., 2021) or gait retraining.

What is more, synergy-based rehabilitation interventions with functional electrical stimulation (FES) have recently emerged, with studies targeting muscle synergies that show temporal delays or poor activation. FES involves applying electrical impulses to activate specific muscles (usually on a single muscle, such as the tibialis anterior, with the use of a single-channel controller), and in the context of stroke rehabilitation, it can be used to enhance gait by improving muscle coordination, promoting more symmetric walking patterns, cope with foot drop, and potentially reinforcing more normative muscle synergies (Howlett et al., 2015). For example, refer to Niu et al. (2019) for a FES intervention on the upper limb of stroke survivors, and to Ferrante et al. (2016) for a targeted intervention on two stroke patients with the aim of mimicking healthy muscle synergies during gait. In the content of this work, abnormal gait muscle synergies can be identified based on similarity metrics between the profiles of stroke patients and healthy populations [e.g., circular cross correlation, time lags, and hindered activation durations; see also (Ferrante et al., 2016)] and inform the activation of multi-channel FES controllers to support tailored gait rehabilitation interventions after stroke. Even though the research on the field is yet very limited, both abovementioned studies indicated promising clinical results for a synergy-based FES intervention toward normal motor function. Finally, musculoskeletal models that export muscle activations may be finally used to determine the optimal number and choice of muscles for muscle synergy extraction in the clinical assessment of stroke patients [similarly to Steele et al. (2013)] and the combined effects of different walking speeds. The same approach may be used to predict the effect of physiotherapy interventions aimed at improving the function of a group of muscles.

## 5 Conclusion

While muscle activation patterns in stroke patients can vary based on the severity of their condition and the time since the ischemic event, stroke survivors in this study displayed a muscle synergy composition during walking that was consistent across both their paretic and non-paretic limbs, as well as with healthy control subjects. This suggests a certain degree of preserved motor function in the affected limbs, even in the presence of recent hemiparesis. Nevertheless, we observed delays and variations in the duration of synergy temporal activations between the groups studied, in the synergies of the hip flexors and knee extensors, dorsal flexors, and plantar flexors. Further optimization of muscle synergy extraction could be achieved by incorporating the activation of additional muscles at different walking speeds. This approach could offer deeper insights into motor function, helping clinicians design rehabilitation strategies that target the activation of specific muscles involved in abnormal synergies, enabling a more individually tailored and outcome-focused approach to stroke rehabilitation. Future research should focus on improving prediction models by incorporating a wider range of muscles and gait speeds to better capture the natural variability of motor control. Additionally, longitudinal studies may track how muscle synergy patterns evolve during rehabilitation, providing valuable data on the plasticity of the neuromuscular system and informing the development of effective and personalized interventions.

## Data Availability

The original contributions presented in this study are included in this article/supplementary material, further inquiries can be directed to the corresponding author.
